# Editorial: Biomarkers in Neurology

**DOI:** 10.3389/fneur.2020.00190

**Published:** 2020-03-18

**Authors:** Stefania Mondello, Mohamed Mosaad Salama, Wael M. Y. Mohamed, Firas H. Kobeissy

**Affiliations:** ^1^Department of Biomedical and Dental Sciences and Morphofunctional Imaging, University of Messina, Messina, Italy; ^2^Institute of Global Health and Human Ecology, American University in Cairo, Cairo, Egypt; ^3^Clinical Pharmacology Department, Menoufia Medical School, Menoufia University, Al Minufya, Egypt; ^4^Department of Basic Medical Science, Kulliyyah of Medicine, International Islamic University, Kuantan, Malaysia; ^5^Department of Emergency Medicine, University of Florida, Gainesville, FL, United States; ^6^Department of Biochemistry and Molecular Genetics, Faculty of Medicine, American University of Beirut, Beirut, Lebanon

**Keywords:** biomarkers, neurological disorders & brain damage, diagnostic test, prognosis, neurodegeneration, brain Injury - Traumatic

Neurological disorders constitute a major health and socioeconomic problem. They represent the second cause of death and the leading cause of disability throughout the world. Despite the implementation of strategies and intervention programs to reduce the burden, over the past 25 years, the incidence, prevalence, mortality, and disability rates of neurological disorders are rising globally, mainly due to population aging and growth (1). This has placed heavy pressure on health-care systems pointing out the urgent need to identify new strategies to improve patient outcomes and reduce health costs by enabling more effective drug development and establishing a more personalized medicine approach.

Rapid scientific and technical advances have enabled reliable and affordable measurement of novel biomarkers—biological indicators that objectively measure and evaluate physiological or pathophysiological processes or pharmacological responses to a therapeutic intervention (2)—which have been suggested to help assessment and management of patients with neurological disorders beyond current practice standards (3–5). Evidence suggests a potential variety of clinical applications, including enhancing diagnostic and prognostic accuracy, improving the existing decision criteria for early diagnosis and risk stratification, as well as assisting in disease monitoring, and acting as surrogate endpoints in experimental studies and clinical trials (6–10). In addition, biomarkers may reliably capture the different aspects of disease heterogeneity and pathogenesis, helping characterize patients, and thereby informing targeted tailored treatments and predicting response outcomes to interventions (11–18). However, despite large numbers of candidate biomarkers have been proposed and extensively evaluated, very few are currently integrated into routine clinical practice and the quest for novel brain injury markers in still ongoing (19).

This book aimed at providing an overview of the biomarker landscape in neurological disorders. The diverse authors discuss established and emerging biomarkers as well as innovative strategies for identifying novel candidates offering new and unique perspectives. Several articles in this volume have been focused on Alzheimer's disease and other neurodegenerative disorders, exploring potentially relevant genetic signature (Chen et al.) and the pathogenetic and prognostic role of circulating cytokines (Kim et al.). Importantly, using a methodologically novel approach that combines computational prediction and experimental validation, Yao et al., for the first time, identified VLDLR, an apolipoprotein E receptor involved in synaptic plasticity, as a circulating signature for Alzheimer's disease. Accordingly, several lines of evidence are pointing toward the added and complementary value of markers of synaptic function owing to their close link with cognitive deterioration (20).

Contemporary investigations on microRNAs (miRNAs) (Di Pietro et al.) and high mobility group box protein 1 (HMGB1) (Paudel et al.) are also presented, highlighting the fact that these markers may be risk factors themselves and therefore potential targets of therapy (21). Diverse contributions recognize the urgent need for reliable diagnostic and prognostic biomarkers in peripheral demyelinating diseases (Kamil et al.) and spinal cord injury (Albayar et al.), with an emphasis on recent advances in medical knowledge and practice; while other work provides an opportunity to study established markers, such as neurofilament light chain, in neonatal neuronal injury (Depoorter et al.), and to demonstrate the theragnostic potential—capability to identify and monitor the drug effect on the molecular pathology—of PAS-positive vacuolated lymphocytes in late-onset Pompe disease patients treated with ERT (Parisi et al.). Finally, the role of lipidomic analysis (Sabogal-Guáqueta et al.) and Fourier-transform infrared imaging spectroscopy and Laser ablation LA-ICPMS techniques (Ali et al.) in biomarker discovery is outlined.

Overall, this volume offers a unique opportunity to foster knowledge and innovation in the arena of biomarkers for neurological disorders, while stimulating testable hypotheses and the development of a strategic research agenda to accelerate their incorporation into routine clinical practice.

## Author Contributions

SM wrote the original draft, assembled and incorporated comments from the co-authors and crafted the final draft. All of the other co-authors contributed to manuscript review and revision.

### Conflict of Interest

The authors declare that the research was conducted in the absence of any commercial or financial relationships that could be construed as a potential conflict of interest.
